# MOTIVATING INFORMATION-SEEKING BEHAVIORS FOR NEW TECHNOLOGY ACROSS THE LIFE SPAN

**DOI:** 10.1093/geroni/igae098.2118

**Published:** 2024-12-31

**Authors:** Li Chu, kayla Patterson, Tasha Kim, Sanjana Srivastava, Ruohan Zhang, Jiajun Wu, Li Fei-Fei, Laura Carstensen

**Affiliations:** Stanford University, Stanford, California, United States; Stanford University, Stanford, California, United States; Stanford University, Stanford, California, United States; Stanford University, Stanford, California, United States; Stanford University, Stanford, California, United States; Stanford University, Stanford, California, United States; Stanford University, Stanford, California, United States; Stanford University, Stanford University, California, United States

## Abstract

Rapid technological advancements demand constant adaptation, which may be particularly unappealing for older adults. As people age, goal priorities shift from exploration to emotional meaning, but modern technology often overlooks this aspect, reducing motivation for older adults to learn. This pre-registered study aims to understand how alignment of information with age-relevant motivation may change people’s information-seeking behaviors. We hypothesized a negative association between age and information-seeking behaviors. Moreover, this association is hypothesized to be moderated by emotional meaning, such that the positive relationship is amplified when technology emphasizes emotional meaning, indicating that older adults are as, if not more, interested in technology compared to younger adults when technology aligns with one’s age-related motivation. We will report findings from an online experiment on a life-span sample of approximately 300 participants. On the data collection website, participants will view eight technological products and choose which ones to explore further. By clicking on the product, a new sentence about the product will be displayed, so the number of clicks and the duration of reading each product may be indices of people’s information-seeking tendencies. Products are randomly assigned to emphasize exploration, social networking, entertainment, or deepening connections with loved ones (the emotionally meaningful option).

